# Prediction of the Potential Host of Peste Des Petits Ruminants Virus by the Least Common Amino Acid Pattern in SLAM Receptor

**DOI:** 10.1155/2024/4374388

**Published:** 2024-04-09

**Authors:** Xin Fan, Arivizhivendhan Kannan Villalan, YeZhi Hu, XiaoDong Wu, HaoNing Wang, XiaoLong Wang

**Affiliations:** ^1^College of Wildlife and Protected Area, Northeast Forestry University, Harbin 150040, Heilongjiang Province, China; ^2^Key Laboratory for Wildlife Diseases and Bio-Security Management of Heilongjiang Province, Harbin 150040, Heilongjiang Province, China; ^3^China Animal Health and Epidemiology Center, Qingdao 266032, Shandong Province, China; ^4^School of Geography and Tourism, Harbin University, Harbin 150086, Heilongjiang Province, China

## Abstract

*Peste-des-Petits Ruminants Virus* (PPRV) causes a highly contagious and severe infectious disease known as *Peste-des-Petits Ruminants* (PPR), resulting in significant mortality in both domestic and wild ruminants. An in-depth understanding of the molecular relationship between PPRV and susceptible hosts is essential for the prevention of PPR. The signaling lymphocytic-activation molecule (SLAM) acts as a key receptor in susceptible host species, mediating interactions with PPRV and triggering PPR in ruminants. This study offers an in-depth analysis of PPRV-susceptible host species as well as the identified SLAM amino acid sequences to date. Investigation reveals that nine families—*Bovidae*, *Camelidae*, *Cervidae*, *Elephantidae*, *Suidae*, *Felidae*, *Canidae*, *Muridae*, and *Ceratopogonidae*—have been affected by PPRV infection. Furthermore, a bioinformatics-based approach was proposed to screen the least common amino acid patterns (LCAP) in important SLAM receptor regions of known PPRV-susceptible species. Research findings reveal that 14 least common amino acid sites (LCAS) in SLAM amino acid sequences (I61, I63, S60, S70, K76, K78, I79, S81, L82, E123, N125, S127, V128, and F131) exhibit a prevalent similarity to LCAP across all known susceptible species. Comparative analysis of these 14 LCAP with SLAM nucleotide sequences from unknown susceptible ruminants to identify species at heightened risk of PPRV. In the result, 48 species from 20 different families across six orders were at potential risk of being infected with PPRV. This exploration suggests the feasibility of assessing potential hosts at high risk of PPRV infection through the LCAS screening technique. Moreover, it offers a means to anticipate and issue warnings regarding the likelihood of interspecies transmission. In conclusion, this study integrates molecular biology and bioinformatics, shedding light on PPRV infection dynamics and paving the way for predictive strategies to prevent the spread of this devastating disease among ruminant populations.

## 1. Introduction

*Peste des Petits Ruminants virus* (PPRV), belonging to the genus *Morbillivirus*, is the causative agent of *Peste des Petits Ruminants* (PPR), a transboundary animal disease affecting a variety of hosts [1–3]. The extensive host range of PPRV poses challenges in disease control strategies, especially when outbreaks involve unusual or novel hosts, highlighting the virus's propensity for inter- and intratransmission. Goats and sheep were the most susceptible of all hosts, and numerous experimental studies have shown that goats were more susceptible than sheep [4, 5]. PPR infection was first reported in 1987 among three wild ruminants such as *Gazella dorcas*, *Capra nubiana*, and *Oryx gazella*. According to epidemiology, interspecific transmission between wild animals and domesticated ruminants was possible due to sharing the same vegetation, water supply, direct or indirect contact, and other resources [6, 7]. The impact of PPRV was profound, causing high mortality rates and significant economic losses in small ruminants [8–10]. PPRV has spread throughout Africa, Asia, the Middle East, and Europe since it was originally discovered in Africa in the 1940s [11, 12]. The World Organization for Animal Health (WOAH) and the Food and Agriculture Organization (FAO) have set a target to control the spread of PPR as the next important zoonotic disease under the Global Eradication Program (GEP) because of its significant threat to the world economy [13]. Elucidating the potential susceptible hosts of PPRV becomes crucial for preventing infection transmission before outbreaks occur. To address this, efforts have been made to consolidate the scattered data on PPR, summarizing information on the susceptibility of a wide range of wildlife species, large ruminants and unusual hosts [14], including goats, sheep, cattle, camels [1, 15], deer and white-tailed deer [16, 17], Asiatic lions [18], pigs [3], Asian elephants [19], dogs [20], and wild small ruminants [21–25].

Viruses initiate infection by recognizing and binding to specific receptors on susceptible host cells. The reliability of establishing a computational method based on the interaction between viruses and receptors to assess and predict the susceptibility of hosts has been demonstrated by numerous studies [26]. The computational approach was used to predict the likelihood of viral transmission between possible hosts based on the theory that species-specific variations in cell-receptor sequences represent the primary barrier to virus infection [27]. The catalytic framework for estimating the force of infection proposes common, external risk variables that impact all species or cross-species transmission [28]. Prediction of virus–receptor interactions using a least-squares algorithm with Laplacian regularization and an initial interaction estimation method via neighbors [29]. Regularized least-squares were utilized in the prediction model to determine possible interactions between the virus and the receptor based on knowledge about the viral and receptor sequences [30]. Many researchers have utilized phylogenetic trees related to the virus–host receptors, exploring divergent or convergent evolutionary branches [24, 31–33]. Computational methods, prediction models, and phylogenetic tree analyses play crucial roles in understanding and predicting susceptibility. However, a clear definition and classification of potential hosts based on receptor strategies remain an area that requires further exploration.

The molecular interactions between PPRV and host cells, such as receptor recognition, adaptation to the host cellular machinery, and evasion of innate immune recognition, determine the host range of PPRV [34]. PPRV specifically interacts with significant receptors of the host [35–37]. The signaling lymphocyte activation molecule (SLAM) is a primary key receptor of the host that significantly promotes the interaction of PPRV glycoprotein [38]. SLAM receptor expression plays a role in post-transcriptional regulation during PPRV infection [39]. It has been established that the SLAM V structural domain's amino acid sites, such as N58–R85, F119–F131, and I210, A211, S226, and R227, were crucial structural domains for interacting with viral proteins and include crucial binding sites for viral proteins [31, 40–45]. The comparison of key amino acid sites in the receptor proves to be an effective approach for predicting potential hosts, thereby enabling proactive measures to prevent disease transmission.

The novelty of our study lies in the specific focus on PPRV and the SLAM receptor, aiming to identify key amino acid site positions that are crucial to the interaction between the virus and its host. Acknowledge the existing gap in knowledge regarding the common features of the genome's uniform classification for PPRV-infected hosts. Despite similarities in the homology amino acid sequences of SLAMs among known PPRV-sensitive species, universal receptor essential amino acid sites for accurately identifying probable hosts are yet to be identified. To address this gap, our research aims to develop fast and accurate computational methods for examining susceptibility to PPRV based on homologous host genomic information.

A unique feature of this study demonstrated the comparative analysis of susceptibility and resistance to goats and another ruminant virus (PPRV), examining these factors at the amino acid level. By using bioinformatics-based techniques, we proposed to classify susceptible species by identifying the least common amino acid sites (LCAS) in the SLAM amino acid sequence and predict potential host species based on matching LCAS with the standard goat SLAM sequence, the process of screening out LCAS called least common amino acid patterns (LCAP). This approach can serve as a guide to identify possible hosts at high risk of infection, offering early warning signals. This research addresses a critical aspect of PPRV infection by focusing on the SLAM receptor and aims to provide a practical tool for identifying potential hosts contributing to the PPR Global Eradication Program to eliminate PPRV.

## 2. Materials and Methods

### 2.1. Collection of PPRV Susceptible Host

All pertinent information on PPRV-infected wild species, domestic species, typical hosts (goats and sheep) or atypical hosts (camels, cattle, deer, etc.), etc. were collected from online databases, including WOAH (https://www.woah.org/en/home/), FAO (https://www.fao.org/home/en), PubMed (https://pubmed.ncbi.nlm.nih.gov/), ScienceDirect (https://www.sciencedirect.com/), and Google Scholars (https://scholar.google.com/), etc. The hosts were summarized by collecting literature and reports of PPRV-infected hosts over the years. The collected information was scrutinized by eliminating the host species that had inappropriate information, such as a lack of temporal and spatial information, literature with duplicate information, non-PPR studies, and other ineligible literature. The PPRV-infected species were classified into two groups based on the source of infection in WOAH's latest Terrestrial Animal Health Code [46]: naturally PPRV-infected species and laboratory PPRV-infected species. Furthermore, they were subclassified into three categories such as clinical surveillance, virological surveillance, and serological surveillance.

### 2.2. Collection of SLAM Amino Acid Sequences

The currently available SLAM amino acid sequences of PPRV-infected and noninfected species from worldwide were collected through the National Center for Biotechnology Information (NCBI), DNA Data Bank of Japan (DDBJ), and The Universal Protein Resource (Uniprot). In further analysis, sequences with 100% identity were excluded from the dataset. The SLAM amino acid sequence of the PPRV-infected goat (GenBank accession No. ABB58752.1) was selected as a typical species for this study.

### 2.3. Multiple Sequences Alignment and Screening LCAS in SLAM Sequence

The SLAM sequences of the collected species were split into two groups, such as the standard SLAM sequence group (known PPRV susceptible host SLAM sequence) and the experimental SLAM sequence group (other species SLAM sequence), according to the PPRV susceptibility. The standard SLAM sequence group was arranged to check the common amino acid sites of all known PPRV susceptible species using multiple sequence alignment. Sequence alignment comparison of standard SALM sequence sets was processed by MAFFT V7.505, with the algorithm using the default parameters in the MEGA X software (http://www.megasoftware.net/). The substitution saturations of all sites were analyzed and transferred to BioeditV7 for editing and JalviewV2.2 for computation (Figure S1). The important PPRV interacting V domain amino acid sites from the SALM sequence were selected according to the previous reports [31, 40–45]. The common amino acid similarity in the V domain SLAM sequence of all the species in the Standard SLAM group was investigated using BioEdit tools. This process involved editing and filtering the sequence data to determine the most conserved and commonly found sites within the standard SLAM group. The most similar sites, referred to as LCAS, were identified from the important V domains, and inconsistent sites were removed.

### 2.4. Prediction of Potentially High-Risk PPRV Susceptible Hosts

**Figure 1:**
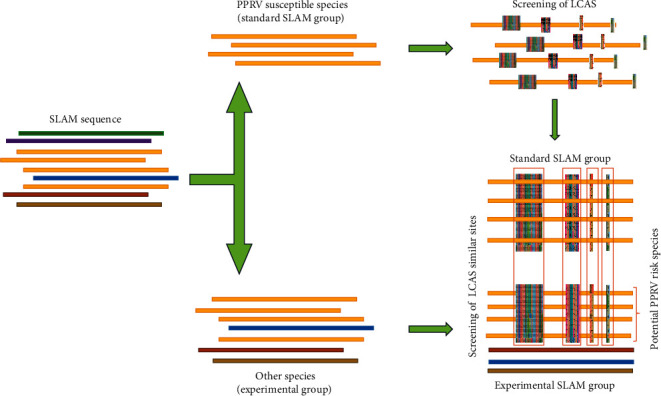
Schematic diagram of prediction of PPRV infection risk host using LACP.

The Standard SLAM sequence groups and experimental SLAM sequence groups were merged individually in MAFFT V7.505 by the imported Standard SLAM sequence group, followed by the experimental SLAM sequence. The LCAS regions of the standard SLAM sequence group were compared with the experimental SLAM sequence group. We define the process of screening out LCAS as LCAP. The potential PPRV risk species were separated from the experimental SLAM sequence group based on the similarity of the LCAP with the standard SALM sequence group. PhyloSuiteV1.2.2 operations were performed on the predicted species based on SLAM and displayed using iTOL V6.7.4 [47]. The detailed process of predicting the potential host for PPRV using LCAS in SLAM sequences is shown in Figure 1.

## 3. Results

### 3.1. PPRV Susceptible Species

**Figure 2:**
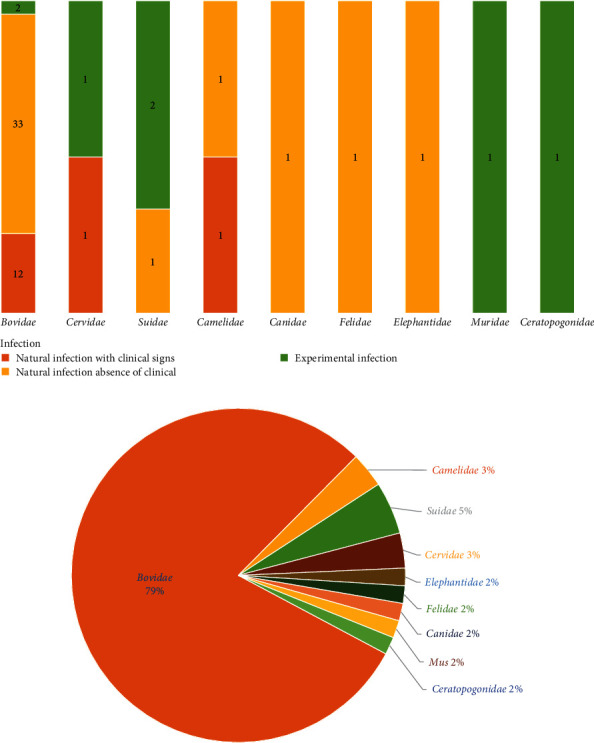
A horizontal comparison of infection types by family can clearly see the accumulation bar chart of the number of reported infection types in every family that has reported infections, (a) with three shades of color indicating the type of infection. (b) The percentage of the total number of reported infected species belonging to each family. The proportion of species belonging to each family can be clearly indicated.

In this study, we collected 59 PPRV-susceptible species documented so far (Table S1). The results reveal that PPRV-infected species across nine families: *Bovidae*, *Camelidae*, *Cervidae*, *Elephantidae*, *Suidae*, *Felidae*, *Canidae*, *Muridae*, and *Ceratopogonidae*. Among them, 14 species were identified through clinical surveillance in a natural source, while 38 were identified through virological surveillance, and 7 were identified through serological surveillance in laboratory conditions (Figure 2(a)). The *Bovidae* had the highest number of PPRV-infected species, with approximately 47 species. *Bovidae* accounted for the largest proportion of 79%, followed by *Suidae* at 5%, *Camelidae* at 3%, *Cervidae* at 3%, and other families at 2% (Figure 2(b)). According to our examination of the literature, clinical symptoms and the presence of antibodies discovered by cELISA were the primary means of diagnosing PPRV infection in the *Cervidae* and *Suidae* species. Species in the *Canidae*, *Felidae*, *Camelidae*, and *Elephantidae* were found to be infected both through the presence of PPRV antigen via cELISA and experimental infection. Species belonging to the *Muridae* family were infected with PPRV during an experimental study, and species from the *Ceratopogonidae* family tested positive for PPRV antigen during the PPRV outbreak (Table S1). PPRV infection was detected in cattle, deer, pigs, camels, and deer, all of which showed clinical symptoms confirmed through antigen, antibody, and serology testing. However, dogs, felines, and elephants did not exhibit any clinical symptoms.

### 3.2. Screening of the Least Common Amino Acid Sites

**Figure 3:**
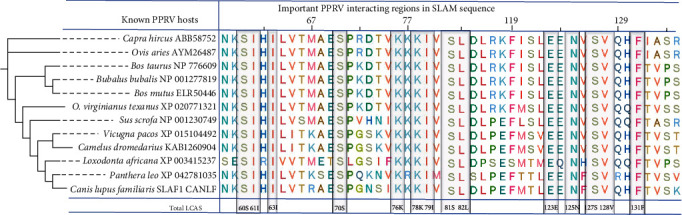
The 45 key domains of the 12 known PPRV-susceptible species, from left to right: N58-R85, F119-F131, I210, A211, S226, R227, on the left side, the full length of their Species name and its phylogenetic tree established by IQ-tree, and displaying through iTol. The LCAS were screened and marked in gray color box.

**Figure 4:**
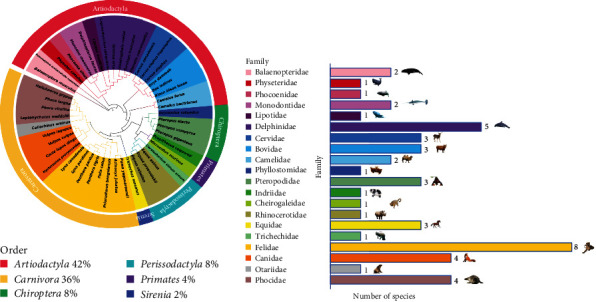
The phylogenetic tree established based on SLAM full-length amino acid sequences will be Phylosuite ModelFinder module to calculate the optimal models suitable for IQ-TREE and then be built. (a) The outer circle represents the order of the species in the total population, and the inner circle represents the species of the family. 42% Artiodactyla, 36% Carnivora, 8% Chiroptera, 8% Perissodactyla, 4% Primates, and 2% Sirenia. (b) The number of species in each family.

The molecular analysis was conducted to predict the potential PPRV risk species. Therefore, the PPRV interacting SLAM sequence was collected from 117 species (Figure S1). Among these, 12 species were reported to be infected with PPRV, and 105 species were not reported for PPRV infection, as summarized in Table S2. The molecular similarity shared among the 12 PPRV-infected species in the standard SLAM sequence group was compared to the 105 species in the experimental SLAM sequence group. Initially, 45 key amino acid domains within the SLAM sequence were screened (Figure 3). Subsequently, 14 important LCASs (I61, I63, S60, S70, K76, K78, I79, S81, L82, E123, N125, S127, V128, and F131) were identified from the SLAM amino acid sequence in 12 species belonging to the standard SLAM sequence group. These LCASs were chosen based on the common similarity of amino acid sites in the standard SLAM sequence group (Figure 4). The 14 important LCAS were separated and used for further investigation in predicting the risk of PPRV susceptibility.

### 3.3. Analysis of Potential Hosts for PPR

The potential PPRV risk species from the experimental SLAM sequence group were identified by their similarity to the 14 important LCAS of the standard SLAM sequence group (Table S2). The findings suggest that 48 species of SLAM sequence displayed complete similarity with the 14 key LCAS of the standard SLAM sequence group. The phylogenetic analysis results revealed that 48 species from 20 different families across six orders (20 species in *Artiodactyla*, 17 species in *Carnivora*, four species in *Chiroptera*, four species in *Perissodactyla*, two species in *Primates*, and one species in *Sirenia*) (Figures 4(a) and 4(b)). In the comprehensive LCAS comparative analysis, it was observed that 42% of species from the *Artiodactyla* order and 36% of species from the *Carnivora* exhibited a significant similarity to the key LCAS of known susceptible species. This was followed by species from orders, such as *Chiroptera* (8%), *Perissodactyla* (8%), *Primates* (4%), and *Sirenia* (2%). Among these, species from the *Artiodactyla* and *Carnivora* orders were more likely to be susceptible to PPRV infection compared to species from other orders, such as *Chiroptera*, *Perissodactyla*, *Primates*, and *Sirenia*.

## 4. Discussion

The host range of PPR in recent years was summarized, and the results demonstrated that ruminants were primarily infected by PPRV, including *Bovidae* and *Cervidae*. Other species, such as pigs, dogs, cats, and elephants, were also likely to be infected. This suggests that the diversity of PPRV-infected species had been expanding and that numerous species might have been at potential risk of PPRV infection. *Bovida*e and *Cervidae* both belong to the ruminant, and recent research has revealed that the ruminant headgear of the bovid family has a common evolutionary origin [48, 49]. It suggests that there was some degree of de genetic correlation between the two. The research demonstrates that white-tailed deer were considerably infected with PPRV [12, 13]; in 1976, laboratory experiments on PPRV infection in white-tailed deer were first conducted in the United States, which confirmed the infection of PPRV in deer. In 2018, natural cases of infection in water deer with obvious clinical symptoms were reported in China, indicating the high risk of PPR infection in deer. Large ruminants such as camels and cattle were considered the dead-end hosts for PPR transmission [50], but their role in the spread of the disease was unknown. PPR infection with clinical symptoms was first reported in camels in 2004, followed by reported cases with clinical symptoms in Iran in 2013 and Kenya in 2016. As a large ruminant, serious PPR infection of camels cannot be ruled out. In SLAM amino acid sequence alignment, the amino acid sequence in the SLAM receptor key domain of the *Bos indicus x Bos taurus* crossbred cattle has undergone a significant change from the original bovine (Table S2). However, at present, bovine have been reported to be infected by PPRV [51], and no cases of PPRV infection have been reported in crossbred cattle, which may be related to the differences in the SLAM receptors of the two. Other feline species differ from PPRV-infected Asiatic lions, although SLAM receptor sequences were consistent with other susceptible hosts [18]. This demonstrates that the susceptibility of large beasts in the cat family to PPR varies significantly and may be linked to the coevolution in different geographical regions. Pigs reported the presence of PPRV antigen and were detected serologically, yet they displayed no clinical symptoms. Positive PPRV results were reported, and PPRV was detected in dogs through both antigenic antibody and serological tests, confirming cross-species transmission. In 2022, laboratory infection experiments on mice showed clinical symptoms, serving as a clear indicator of PPR infection in mammals. This suggests the necessity to expand the warning range of PPR.

Recent research demonstrated that important cell receptor loci were strongly associated with the propensity of viruses to invade hosts. The SLAM receptors are important cellular receptors for measles viruses such as rinderpest virus, morbillivirus, and canine distemper virus [52]. In 2003, Ohno et al. [31] demonstrated that the 61st histidine and its neighboring amino acid residues were critical to SLAM's (CD150) ability as a cellular receptor for the measles virus. In 2004, Hu et al. [40] confirmed that amino acids at positions 27–135 in SLAM exhibited the highest interaction activity with PPRV H through yeast two-hybrid experiments. In 2008, Ohishi et al. [41] predicted host-virus specificity of the measles virus through structural modeling of SLAM receptor in Marine mammals, showing that eight amino acid residues (64, 67, 69, 73, 85, 119, 121, and 130) at position 58–130 determined host-virus specificity. These animal populations were sensitive to PPRV. Amino acid residues at positions 58–63, 210−211, and 226–227 of human and sheep SLAM proteins played a key role in the SLAM receptor function of PPRV and MV [31, 40, 42]. In 2016, Liang et al. [43] found that certain residues in SLAM (62–82, 123, and 127–131) were crucial for determining its binding potential in both sheep and humans, using molecular docking. In 2020, Meng et al. [44] discovered that specific amino acid residues played a key role in the interaction between PPRV H and cell receptors. Amino acids I61, H62, L64, K76, K78, E123, H130, I210, A211, S226, and R227 in SLAM were found to be crucial for the specificity of H-SLAM interactions. PRV shared similar antigenic features with the mentioned viruses, allowing them to cross species barriers. They also had a similar mechanism for transmitting between species and adapting to new hosts [53–55]. SLAM is the principal active receptor for PPRV binding to host cells, and many studies have demonstrated that SLAM was closely related to host susceptibility and species specificity of PPRV infection [31, 56, 57]. Moreover, Nectin-4 involves an epithelial receptor with less interaction with the host specificity of PPRV [58].

We identified the most important 14 key LCASs in the SLAM receptor sequence from the known PPRV susceptible species. Based on the sequence similarity of 14 key LCASs, we predicted 48 potential host species in 20 families of unknown susceptible species. The SLAM receptor sequences of 48 species were commonly matched with 14 LCASs of known PPRV-susceptible species. Therefore, the SLAM receptor cells from these 48 species were highly likely to interact with PPRV and lead to infection. The main objective of this study was to eliminate the amino acid sites that are uncommon in known PPRV-susceptible hosts using the LCAP. It allowed us to draw the initial conclusion that these sites do not play a crucial role. The list of prospective hosts was aggressively broadened for inclusion, starting with the least common sites that were the most precise. Combining macroscopic and microscopic elements that were essential for wildlife conservation helped us ascertain the practical importance of the anticipated results in the virus's spread. The least common amino acid rule should be used to determine the susceptibility of species to PPR, taking into account a variety of realistic factors like activity space, habits, and feeding relationships between conspecifics.

However, due to the large number of species in each family, this work has certain limitations. Our study was done on a limited number of SLAM receptors in the species that have been reported so far, and only the differences in amino acids between species were considered here, so there may be, as yet, undiscovered host species with different SLAM receptor configurations. Since the method cannot guarantee the accuracy of sensitivity for each species, which may increase the likelihood of inclusion of non-potential hosts. Hence, laboratory studies are necessary to validate the basis and visualize the molecular docking results through 3D modeling, which needs to be verified by the further experimental study. Additionally, various factors influence PPRV transmission across species, including host immunological response, which may also contribute to vulnerability. Therefore, in order to keep the PPRV eradication program on track and prevent spillover, it is necessary to predict potential hosts with the highest degree of confidence using currently available information. This approach will also help ensure that no potential PPRV-infected species are missed, thereby helping to protect biodiversity.

## 5. Conclusion

This study significantly expands the understanding of PPRV-infected hosts through targeted screening of common amino acid sites. By assessing the LCAS similarity of the major SLAM receptor regions in a known PPRV-sensitive host, we have delineated the potential host range of PPRV. The findings of this study offer practical insights for identifying hosts crucial for the future prevention and eradication of PPRV. The potential host for PPRV should be continuously monitored by various methods to control the spread of PPRV to new hosts. This method's versatility will enable advancements in other fields to strengthen animal disease control and surveillance, which was the method's original intent. This method provides a favorable framework for interpreting and addressing various potential PPRV infection risk animals for PPRV. This study provides a basic blueprint for monitoring and controlling future instances of interspecies transmission.

## Data Availability

The experimental data used to support the findings of this study are available from the corresponding author upon request.

## Supplementary Materials

Supplementary Materials Figure S1: alignment process of SALM sequences from both standared and experimental group. Table S1: species that have been reported to be infected with PPRV. Table S2: SLAM amino acid sequence summary.
