# Supplementary material for: Prediction of the Potential Host of Peste Des Petits Ruminants Virus by the Least Common Amino Acid Pattern in SLAM Receptor
Source: Transbound Emerg Dis. 2024 Apr 9;2024:4374388. doi: 10.1155/2024/4374388 (PMC12017033; doi:10.1155/2024/4374388)
Supplement: Supplementary Materials — Figure S1: alignment process of SALM sequences from both standared and experimental group. Table S1: species that have been reported to be infected with PPRV. Table S2: SLAM amino acid sequence summary. [file 4374388.f1.docx]

**Transboundary and Emerging Diseases**

**Prediction of the potential host of Peste des Petits Ruminants virus by the least common amino acid pattern in SLAM receptor**

Xin Fan,1,2 Arivizhivendhan Kannan Villalan,1,2 YeZhi Hu, 1,2 XiaoDong Wu, 3 HaoNing Wang, 4 and XiaoLong Wang 1,2*

1 College of Wildlife and Protected Area, Northeast Forestry University, Harbin 150040, Heilongjiang province, P. R. China

2 Key Laboratory for Wildlife Diseases and Bio-security Management of Heilongjiang Province, Harbin 150040, Heilongjiang province, P. R. China.

3 China Animal Health and Epidemiology Center, Qingdao 266032, Shandong province, P. R. China.

4 School of Geography and Tourism, Harbin University, Harbin University, Harbin 150086, Heilongjiang province, P. R. China.

Correspondence should be addressed to XiaoDong Wu; [wuxiaodong@cahec.cn](mailto:wuxiaodong@cahec.cn), HaoNing Wang; [wanghaoning1017@126.com](mailto:wanghaoning1017@126.com), XiaoLong Wang; [nefuwxl@hotmail.com](mailto:nefuwxl@hotmail.com)

**Supplementary Materials**

Supplementary table 1： Species that have been reported to be infected with PPRV

| Species | Latin name | Country | Year | Study |  |
| --- | --- | --- | --- | --- | --- |
| **Natural PPR infection with clinical signs** | | | | | |
| ***Bovidae*** | | | | | |
| Dorcas gazelles | *Gazella dorcas* | Sudan | 2017 | [1] |  |
|  | *Gazella dorcas* | UAE, KSA | 1987 | [2] |  |
|  | *Gazella dorcas* | KSA | 2004 | [3] |  |
|  | *Gazella dorcas* | Nigeria | 2015 | [4] |  |
|  | *Gazella dorcas* | Sudan | 2016 | [5] |  |
| Mongolian gazelles | *Procapra gutturosa* | China | 2007 | [6] |  |
| Dama gazelle | *Nanger dama* | UAE | 2021 | [7] |  |
| Chowsingha | *Tetracerus quadricornis* | India | 2017 | [8] |  |
| Thompson’s gazelle | *Gazella thomsoni* | KSA | 2004 | [3] |  |
|  | *Gazella thomsoni* | Tanzania | 2016 | [9] |  |
|  | *Gazella thomsoni* | Tanzania | 2014 | [10] |  |
| Tibetan antelope | *Pantholops hodgsonii* | China | 2007 | [6] |  |
| Bezoar ibex | *Capra aegagrus* | Kurdistan | 2010 | [11] |  |
| Sindh ibex | *Capra aegagrus blythi* | Pakistan | 2009 | [12] |  |
| Nubian Ibex | *Capra nubiana* | Arabian Gulf | 1987 | [2] |  |
|  | *Capra nubiana* | UAE | 2009 | [13] |  |
| Wild ibex | *Capra ibex* | China | 2015 | [14] |  |
| Gemsbok | *Oryx gazella* | UAE | 1987 | [2] |  |
|  | *Oryx gazella* | Qatar | 2016 | [15] |  |
| Laristan sheep | *Ovis orientalis laristanica* | India | 2009 | [16] |  |
| ***Camelidae*** |  |  |  |  |  |
| Camels | *Camelus dromedarius* | Sudan | 2004 | [17] |  |
|  | *Camelus dromedarius* | Iran | 2013 | [18] |  |
|  | *Camelus dromedarius* | Kenya | 2016 | [19] |  |
|  | *Camelus dromedarius* | Kenya | 2018 | [20] |  |
| ***Cervidae*** | | | | | |
| Water deer | *Hydropotes inermis* | China | 2018 | [21] |  |
| **Natural PPR infection absence of clinical. Virological monitoring.** | | | | | |
| ***Bovidae*** | | | | | |
| Wildebeest | *Connochaetes gnou* | Tanzania | 2015 | [9] |  |
| Blue wildebeest | *Connochaetes taurinus* | Tanzania | 2014 | [10] |  |
| Hartebeest | *Alcelaphus buselaphus* | Côte d’Ivoire | 2005 | [22] |  |
|  | *Alcelaphus buselaphus* | Tanzania | 2015 | [9] |  |
| Uganda kob | *Kobus kob thomasi* | Uganda | 2015 | [9] |  |
| Kob | *Kobus kob* | Côte d’Ivoire | 2005 | [23] |  |
| Topi | *Damaliscus lunatus* | South Sudan | 2013 | [9] |  |
| Defassa waterbuck | *Kobus ellipsiprymnus defassa* | Qatar | 2016 | [15] |  |
|  | *Kobus ellipsiprymnus defassa* | Côte d’Ivoire | 2005 | [23] |  |
| Waterbuck | *Kobus ellipsiprymnus* | Côte d’Ivoire | 2005 | [23] |  |
| Bushbuck | *Tragelaphus scriptus* | UAE | 2009 | [13] |  |
| Blackbuck | *Antilope cervicapra* | Qatar | 2018 | [15] |  |
| Springbok | *Antidorcas marsupialis* | Qatar | 2019 | [15] |  |
|  | *Antidorcas marsupialis* | UAE | 2009 | [13] |  |
| Impala | *Aepyceros melampus* | Tanzania | 2014 | [10] |  |
|  | *Aepyceros melampus* | Kenya | 2016 | [9] |  |
|  | *Aepyceros melampus* | Tanzania | 2016 | [9] |  |
|  | *Aepyceros melampus* | UAE | 2009 | [13] |  |
| Rheem gazelles | *Gazella subguttorosa marica* | UAE | 2009 | [13] |  |
| Goitered gazelle | *Gazella subgutturosa subgutturosa* | China | 2013 | [24] |  |
|  | *Gazella subgutturosa subgutturosa* | Turkey | 2010 | [25] |  |
| Arabian mountain gazelles | *Gazella gazella cora* | UAE | 2009 | [13] |  |
| Arabian gazelles | *Gazella gazella* | UAE | 2009 | [13] |  |
| Grant’s gazelles | *Nanger granti* | Kenya | 2016 | [9] |  |
|  | *Nanger granti* | Tanzania | 2015 | [9] |  |
|  | *Nanger granti* | Tanzania | 2016 | [9] |  |
|  | *Nanger granti* | Tanzania | 2014 | [10] |  |
| Przewalski's gazelle | *Procapra przewalskii* | Gansu,China | 2018 | [26] |  |
| Gerenuk | *Litocranius walleri* | Kenya | 2016 | [9] |  |
|  | *Litocranius walleri* | Kenya | 2017 | [9] |  |
| Barbary sheep | *Ammotragus lervia* | UAE | 2009 | [13] |  |
|  | *Ammotragus larvia* | UAE | 2021 | [27] |  |
| Argali | *Ovis ammon* | China | 2013 | [24] |  |
| African grey duiker | *Sylvicapra grimmia* | Côte d’Ivoire | 2004 | [23] |  |
|  | *Sylvicapra grimmia* | Nigeria | 2003 | [28] |  |
| Afghan Markhor goat | *Capra falconeri* | UAE | 2009 | [13] |  |
| Addax | *Addax nasomaculatus* | Qatar | 2017 | [15] |  |
| Capra ibex | *Capra ibex sibirica* | China | 2013 | [24] |  |
| Mongolian saiga | *Saiga tatarica mongolica* | Mongolia | 2017 | [29] |  |
|  | *Syncerus caffer* | Tanzania | 2015 | [9] |  |
|  | *Syncerus caffer* | Tanzania | 2016 | [9] |  |
|  | *Syncerus caffer* | Côte d’Ivoire | 2005 | [23] |  |
| Bharals | *Pseudois nayaur* | China | 2012 | [30] |  |
| Nile lechwe | *Kobus megaceros* | Sudan | 2008 | [31] |  |
| Bovine | *Bos grunniens* | Pakistan | 2019 | [32] |  |
| Aurochs | *Bos primigenius taurus* | Tanzania | 2016 | [33] |  |
| African buffalo | *Syncerus caffer* | Kenya | 2016 | [9] |  |
| Indian buffalo | *Bubalus bubalus* | India | 2008 | [34] |  |
|  | *Bubalus bubalus* | Pakistan | 2008 | [35] |  |
|  | *Bubalus bubalus* | India | 2009 | [36] |  |
|  | *Bubalus bubalus* | Vietnam | 2007 | [34] |  |
| Wild yak | *Bos mutus* | China | 2018 | [26] |  |
| ***Elephantidae*** | | | | | |
| Elephant | *Loxodonta africana* | South Sudan | 2013 | [9] |  |
| ***Suidae*** | | | | | |
| Warthog | *Phacochoerus africanus* | Kenya | 2016 | [9] |  |
| ***Camelidae*** | | | | | |
| Alpacas | *Vicugna pacos* | Shanxi,China | 2020 | [37] |  |
| ***Felidae*** | | | | | |
| Asiatic lion | *Panthera leo persica* | India | 2010 | [38] |  |
| ***Canidae*** | | | | | |
| Dog | *Canis lupus familiaris* | Mathura | 2015 | [39] |  |
| **Experimental Infection** | | | | | |
| ***Bovidae*** | | | | | |
| German Edelziege goats | *Capra hircus* | Germany | 2021 | [27] |  |
| West African  dwarf goats | *Capra hircus breed West African Dwarf* | African | 2007 | [40] |  |
| ***Suidae*** |  |  |  |  |  |
| Wild boar | *Sus scrofa* | Germany | 2017 | [41] |  |
| Domestic pigs | *Sus scrofa domestica* | Germany | 2017 | [41] |  |
| ***Cervidae*** |  |  |  |  |  |
| White-tailed Deer | *Odocoileus virginianus* | USA | 1976 | [42] |  |
| ***Muridae*** |  |  |  |  |  |
| Mice | *Mus musculus* | Ireland | 2002 | [43] |  |
| ***Ceratopogonidae*** | | | | | |
| Biting midge | *Culicoides imicola* | Turkey | 2015 | [44] |  |

Supplementary figure 1a & b: Alignment process of SALM sequences from both standared and experimental group. MAFFT comparison and alignment of the SLAM amino acid sequences of goats as a standard to process the other SALM sequences and it’s calculated by JalviewV2.2 software to present Conservation, Quality, Consensus, Occupancy.


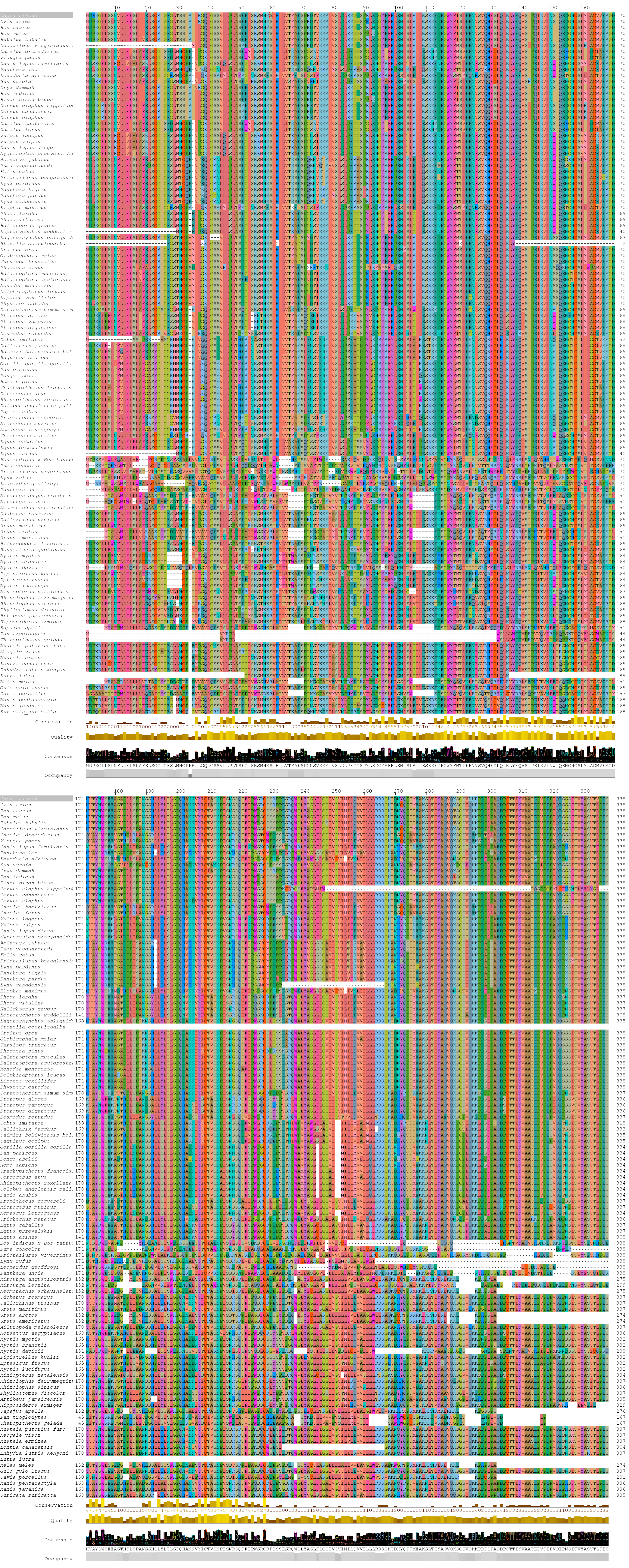


**(a)**

**(b)**


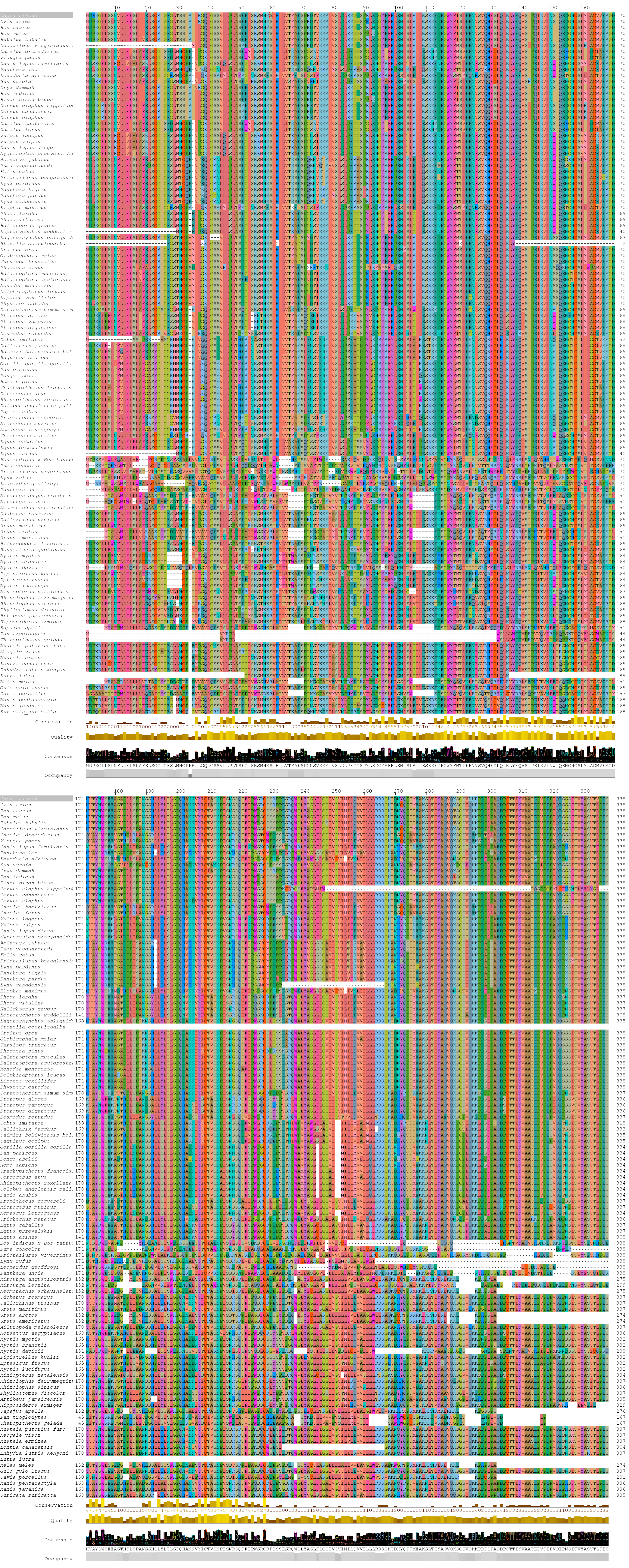


Supplementary table 2: SLAM amino acid sequence Summary. SLAM sequences of known PPRV susceptible species (rows in the table are numbered from 1 to 12) and other species. SLAM sequences of LACS were compared with those of other species, and potential risk hosts of PPRV that were identical to LACS were highlighted in bold.

| No. | Order | Latin name | GenBank LOCUS | 60 | 61 | 63 | 70 | 76 | 78 | 79 | 81 | 82 | 123 | 125 | 127 | 128 | 131 |
| --- | --- | --- | --- | --- | --- | --- | --- | --- | --- | --- | --- | --- | --- | --- | --- | --- | --- |
| 1 | *Artiodactyla* | ***Capra hircus*** | **ABB58752.1** | **S** | **I** | **I** | **S** | **K** | **K** | **I** | **S** | **L** | **E** | **N** | **S** | **V** | **F** |
| 2 | *Artiodactyla* | ***Ovis aries*** | AYM26487 | **S** | **I** | **I** | **S** | **K** | **K** | **I** | **S** | **L** | **E** | **N** | **S** | **V** | **F** |
| 3 | *Artiodactyla* | ***Bos taurus*** | NP_776609.2 | **S** | **I** | **I** | **S** | **K** | **K** | **I** | **S** | **L** | **E** | **N** | **S** | **V** | **F** |
| 4 | *Artiodactyla* | ***Bos mutus*** | ELR50446.1 | **S** | **I** | **I** | **S** | **K** | **K** | **I** | **S** | **L** | **E** | **N** | **S** | **V** | **F** |
| 5 | *Artiodactyla* | ***Bubalus bubalis*** | NP_001277819.1 | **S** | **I** | **I** | **S** | **K** | **K** | **I** | **S** | **L** | **E** | **N** | **S** | **V** | **F** |
| 6 | *Artiodactyla* | ***Odocoileus virginianus***  ***texanus*** | XP_020771321.1 | **S** | **I** | **I** | **S** | **K** | **K** | **I** | **S** | **L** | **E** | **N** | **S** | **V** | **F** |
| 7 | *Artiodactyla* | ***Sus scrofa*** | NP_001230749.1 | **S** | **I** | **I** | **S** | **K** | **K** | **I** | **S** | **L** | **E** | **N** | **S** | **V** | **F** |
| 8 | *Artiodactyla* | ***Camelus dromedarius*** | KAB1260904.1 | **S** | **I** | **I** | **S** | **K** | **K** | **I** | **S** | **L** | **E** | **N** | **S** | **V** | **F** |
| 9 | *Artiodactyla* | ***Vicugna pacos*** | XP_015104492.1 | **S** | **I** | **I** | **S** | **K** | **K** | **I** | **S** | **L** | **E** | **N** | **S** | **V** | **F** |
| 10 | *Carnivora* | ***Canis lupus familiaris*** | Q95MM9.1 | **S** | **I** | **I** | **S** | **K** | **K** | **I** | **S** | **L** | **E** | **N** | **S** | **V** | **F** |
| 11 | *Carnivora* | ***Panthera leo*** | XP_042781035.1 | **S** | **I** | **I** | **S** | **K** | **K** | **I** | **S** | **L** | **E** | **N** | **S** | **V** | **F** |
| 12 | *Proboscidea* | ***Loxodonta africana*** | XP_003415237.1 | **S** | **I** | **I** | **S** | **E** | **E** | **I** | **S** | **L** | **E** | **N** | **S** | **V** | **F** |
| 13 | *Artiodactyla* | ***Oryx dammah*** | XP_040086027.1 | **S** | **I** | **I** | **S** | **K** | **K** | **I** | **S** | **L** | **E** | **N** | **S** | **V** | **F** |
| 14 | *Artiodactyla* | ***Bos indicus*** | ABB58750.1 | **S** | **I** | **I** | **S** | **K** | **K** | **I** | **S** | **L** | **E** | **N** | **S** | **V** | **F** |
| 15 | *Artiodactyla* | ***Bison bison bison*** | XP_010836155.1 | **S** | **I** | **I** | **S** | **K** | **K** | **I** | **S** | **L** | **E** | **N** | **S** | **V** | **F** |
| 16 | *Artiodactyla* | ***Globicephala melas*** | XP_030697940.1 | **S** | **I** | **I** | **S** | **K** | **K** | **I** | **S** | **L** | **E** | **N** | **S** | **V** | **F** |
| 17 | *Artiodactyla* | ***Balaenoptera acutorostrata***  ***scammoni*** | XP_007171815.1 | **S** | **I** | **I** | **S** | **K** | **K** | **I** | **S** | **L** | **E** | **N** | **S** | **V** | **F** |
| 18 | *Artiodactyla* | ***Balaenoptera musculus*** | XP_036719389 | **S** | **I** | **I** | **S** | **K** | **K** | **I** | **S** | **L** | **E** | **N** | **S** | **V** | **F** |
| 19 | *Artiodactyla* | ***Lipotes vexillifer*** | XP_007467163.1 | **S** | **I** | **I** | **S** | **K** | **K** | **I** | **S** | **L** | **E** | **N** | **S** | **V** | **F** |
| 20 | *Artiodactyla* | ***Physeter catodon*** | XP_007124119.3 | **S** | **I** | **I** | **S** | **K** | **K** | **I** | **S** | **L** | **E** | **N** | **S** | **V** | **F** |
| 21 | *Artiodactyla* | ***Tursiops truncatus*** | XP_004327894.1 | **S** | **I** | **I** | **S** | **K** | **K** | **I** | **S** | **L** | **E** | **N** | **S** | **V** | **F** |
| 22 | *Artiodactyla* | ***Monodon monoceros*** | XP_029082620.1 | **S** | **I** | **I** | **S** | **K** | **K** | **I** | **S** | **L** | **E** | **N** | **S** | **V** | **F** |
| 23 | *Artiodactyla* | ***Orcinus orca*** | NP_001266738.1 | **S** | **I** | **I** | **S** | **K** | **K** | **I** | **S** | **L** | **E** | **N** | **S** | **V** | **F** |
| 24 | *Artiodactyla* | ***Lagenorhynchus obliquidens*** | BAH10670.1 | **S** | **I** | **I** | **S** | **K** | **K** | **I** | **S** | **L** | **E** | **N** | **S** | **V** | **F** |
| 25 | *Artiodactyla* | ***Cervus elaphus hippelaphus*** | OWK04952.1 | **S** | **I** | **I** | **S** | **K** | **K** | **I** | **S** | **L** | **E** | **N** | **S** | **V** | **F** |
| 26 | *Artiodactyla* | ***Cervus elaphus*** | XP_043734628.1 | **S** | **I** | **I** | **S** | **K** | **K** | **I** | **S** | **L** | **E** | **N** | **S** | **V** | **F** |
| 27 | *Artiodactyla* | ***Delphinapterus leucas*** | XP_022415222.1 | **S** | **I** | **I** | **S** | **K** | **K** | **I** | **S** | **L** | **E** | **N** | **S** | **V** | **F** |
| 28 | *Artiodactyla* | ***Camelus bactrianus*** | XP_010953950.1 | **S** | **I** | **I** | **S** | **K** | **K** | **I** | **S** | **L** | **E** | **N** | **S** | **V** | **F** |
| 29 | *Artiodactyla* | ***Camelus ferus*** | XP_014417521.2 | **S** | **I** | **I** | **S** | **K** | **K** | **I** | **S** | **L** | **E** | **N** | **S** | **V** | **F** |
| 30 | *Artiodactyla* | ***Cervus canadensis*** | XP_043307596.1 | **S** | **I** | **I** | **S** | **K** | **K** | **I** | **S** | **L** | **E** | **N** | **S** | **V** | **F** |
| 31 | *Artiodactyla* | ***Stenella coeruleoalba*** | ATB24532.1 | **S** | **I** | **I** | **S** | **K** | **K** | **I** | **S** | **L** | **E** | **N** | **S** | **V** | **F** |
| 32 | *Artiodactyla* | ***Phocoena sinus*** | XP_032464762.1 | **S** | **I** | **I** | **S** | **K** | **N** | **I** | **S** | **L** | **E** | **N** | **S** | **V** | **F** |
| 33 | *Carnivora* | ***Callorhinus ursinus*** | XP_025716439.1 | **S** | **I** | **I** | **S** | **K** | **K** | **I** | **S** | **L** | **E** | **N** | **S** | **V** | **F** |
| 34 | *Carnivora* | ***Phoca largha*** | BAH10672.1 | **S** | **I** | **I** | **S** | **K** | **K** | **I** | **S** | **L** | **E** | **N** | **S** | **V** | **F** |
| 35 | *Carnivora* | ***Halichoerus grypus*** | XP_035940608.1 | **S** | **I** | **I** | **S** | **K** | **K** | **I** | **S** | **L** | **E** | **N** | **S** | **V** | **F** |
| 36 | *Carnivora* | ***Phoca vitulina*** | XP_032253137.1 | **S** | **I** | **I** | **S** | **K** | **K** | **I** | **S** | **L** | **E** | **N** | **S** | **V** | **F** |
| 37 | *Carnivora* | ***Vulpes lagopus*** | XP_041578543.1 | **S** | **I** | **I** | **S** | **K** | **K** | **I** | **S** | **L** | **E** | **N** | **S** | **V** | **F** |
| 38 | *Carnivora* | ***Vulpes vulpes*** | ACD47119.1 | **S** | **I** | **I** | **S** | **K** | **K** | **I** | **S** | **L** | **E** | **N** | **S** | **V** | **F** |
| 39 | *Carnivora* | ***Canis lupus dingo*** | XP_025276562.1 | **S** | **I** | **I** | **S** | **K** | **K** | **I** | **S** | **L** | **E** | **N** | **S** | **V** | **F** |
| 40 | *Carnivora* | ***Nyctereutes procyonoides*** | CAD7679895.1 | **S** | **I** | **I** | **S** | **K** | **K** | **I** | **S** | **L** | **E** | **N** | **S** | **V** | **F** |
| 41 | *Carnivora* | ***Acinonyx jubatus*** | XP_026904340.1 | **S** | **I** | **I** | **S** | **K** | **K** | **I** | **S** | **L** | **E** | **N** | **S** | **V** | **F** |
| 42 | *Carnivora* | ***Felis catus*** | NP_001265755.1 | **S** | **I** | **I** | **S** | **K** | **K** | **I** | **S** | **L** | **E** | **N** | **S** | **V** | **F** |
| 43 | *Carnivora* | ***Prionailurus bengalensis*** | XP_043423731.1 | **S** | **I** | **I** | **S** | **K** | **K** | **I** | **S** | **L** | **E** | **N** | **S** | **V** | **F** |
| 44 | *Carnivora* | ***Lynx pardinus*** | VFV35259.1 | **S** | **I** | **I** | **S** | **K** | **K** | **I** | **S** | **L** | **E** | **N** | **S** | **V** | **F** |
| 45 | *Carnivora* | ***Leptonychotes weddellii*** | XP_006743503.2 | **S** | **I** | **I** | **S** | **K** | **K** | **I** | **S** | **L** | **E** | **N** | **S** | **V** | **F** |
| 46 | *Carnivora* | ***Puma yagouaroundi*** | XP_040308375.1 | **S** | **I** | **I** | **S** | **K** | **K** | **I** | **S** | **L** | **E** | **N** | **S** | **V** | **F** |
| 47 | *Carnivora* | ***Panthera tigris*** | XP_007092436.1 | **S** | **I** | **I** | **S** | **K** | **K** | **I** | **S** | **L** | **E** | **N** | **S** | **V** | **F** |
| 48 | *Carnivora* | ***Panthera pardus*** | XP_019288879.1 | **S** | **I** | **I** | **S** | **K** | **K** | **I** | **S** | **L** | **E** | **N** | **S** | **V** | **F** |
| 49 | *Carnivora* | ***Lynx canadensis*** | XP_030157724.1 | **S** | **I** | **I** | **S** | **K** | **K** | **I** | **S** | **L** | **E** | **N** | **S** | **V** | **F** |
| 50 | *Chiroptera* | ***Desmodus rotundus*** | XP_024427094.1 | **S** | **I** | **I** | **S** | **K** | **K** | **I** | **S** | **L** | **E** | **N** | **S** | **V** | **F** |
| 51 | *Chiroptera* | ***Pteropus vampyrus*** | XP_011371357.1 | **S** | **I** | **I** | **S** | **K** | **K** | **I** | **S** | **L** | **E** | **N** | **S** | **V** | **F** |
| 52 | *Chiroptera* | ***Pteropus giganteus*** | XP_039695881.1 | **S** | **I** | **I** | **S** | **K** | **K** | **I** | **S** | **L** | **E** | **N** | **S** | **V** | **F** |
| 53 | *Chiroptera* | ***Pteropus alecto*** | XP_006922948.1 | **S** | **I** | **I** | **S** | **K** | **K** | **I** | **S** | **L** | **E** | **N** | **S** | **V** | **F** |
| 54 | *Perissodactyla* | ***Ceratotherium simum simum*** | XP_004442857.1 | **S** | **I** | **I** | **S** | **K** | **K** | **I** | **S** | **L** | **E** | **N** | **S** | **V** | **F** |
| 55 | *Perissodactyla* | ***Equus caballus*** | XP_001504489.1 | **S** | **I** | **I** | **S** | **K** | **K** | **I** | **S** | **L** | **E** | **N** | **S** | **V** | **F** |
| 56 | *Perissodactyla* | ***Equus przewalskii*** | XP_008506477.1 | **S** | **I** | **I** | **S** | **K** | **K** | **I** | **S** | **L** | **E** | **N** | **S** | **V** | **F** |
| 57 | *Perissodactyla* | ***Equus asinus*** | XP_044614007.1 | **S** | **I** | **I** | **S** | **K** | **K** | **I** | **S** | **L** | **E** | **N** | **S** | **V** | **F** |
| 58 | *Primates* | ***Propithecus coquereli*** | XP_012494990.1 | **S** | **I** | **I** | **S** | **K** | **K** | **I** | **S** | **L** | **E** | **N** | **S** | **V** | **F** |
| 59 | *Primates* | ***Microcebus murinus*** | XP_012604678.1 | **S** | **I** | **I** | **S** | **K** | **K** | **I** | **S** | **L** | **E** | **N** | **S** | **V** | **F** |
| 60 | *Sirenia* | ***Trichechus manatus*** | BAH10674.1 | **S** | **I** | **I** | **S** | **K** | **K** | **I** | **S** | **L** | **E** | **N** | **S** | **V** | **F** |
| 61 | *Primates* | *Pongo abelii* | XP_002809959.2 | S | I | I | S | E | K | I | S | L | E | N | S | V | F |
| 62 | *Primates* | *Nomascus leucogenys* | XP_030679064.1 | S | I | I | S | E | K | I | S | L | E | N | S | V | F |
| 63 | *Primates* | *Trachypithecus francoisi* | XP_033053042.1 | S | I | I | S | E | K | I | S | L | E | N | S | V | F |
| 64 | *Primates* | *Cercocebus atys* | XP_011922993.1 | S | I | I | S | E | K | I | S | L | E | N | S | V | F |
| 65 | *Primates* | *Rhinopithecus roxellana* | XP_010359038.1 | S | I | I | S | E | K | I | S | L | E | N | S | V | F |
| 66 | *Primates* | *Papio anubis* | XP_031510478.1 | S | I | I | S | E | K | I | S | L | E | N | S | V | F |
| 67 | *Proboscidea* | *Elephas maximus* | BAH10675.1 | S | I | I | S | E | E | I | S | L | E | N | S | V | F |
| 68 | *Primates* | *Gorilla gorilla* | XP_004027768.1 | S | I | I | S | E | K | I | S | L | E | N | S | V | F |
| 69 | *Primates* | *Pan paniscus* | XP_034794404.1 | S | I | I | S | E | K | I | S | L | E | N | S | V | F |
| 70 | *Primates* | *Homo sapiens* | NP_003028.1 | S | I | I | S | E | K | I | S | L | E | N | S | V | F |
| 71 | *Primates* | *Colobus angolensis*  *palliatus* | XP_011813018.1 | S | I | I | S | E | K | I | S | L | E | N | S | V | F |
| 72 | *Primates* | *Saguinus oedipus* | AAG18445.1 | S | I | I | S | E | K | I | S | L | E | N | S | V | F |
| 73 | *Primates* | *Callithrix jacchus* | XP_002760222.2 | S | I | I | S | E | K | I | S | L | E | N | S | V | F |
| 74 | *Primates* | *Saimiri boliviensis*  *boliviensis* | XP_003938005.1 | S | I | I | S | E | K | I | S | L | E | N | S | V | F |
| 75 | *Primates* | *Cebus imitator* | XP_037585539.1 | S | I | I | S | E | K | I | S | L | E | N | S | V | F |
| 76 | *Carnivora* | *Odobenus rosmarus* | BAH10673 | S | I | I | S | K | K | V | S | L | E | N | S | V | F |
| 77 | *Chiroptera* | *Rousettus aegyptiacus* | KAF6398003.1 | S | I | I | S | K | K | I | S | L | E | N | S | V | S |
| 78 | *Carnivora* | *Gulo gulo luscus* | KAI5758423.1 | S | V | I | S | K | K | I | S | L | E | N | S | V | F |
| 79 | *Chiroptera* | *Phyllostomus discolor* | XP_035870954.1 | R | I | I | S | K | K | I | S | L | E | N | S | V | F |
| 80 | *Carnivora* | *Neogale vison* | ACM90097.1 | S | V | V | S | K | K | I | S | L | E | N | S | V | F |
| 81 | *Carnivora* | *Mustela putorius furo* | XP_004775935.1 | S | V | V | S | K | K | I | S | L | E | N | S | V | F |
| 82 | *Carnivora* | *Ursus maritimus* | XP_008698905.1 | S | T | V | S | K | K | I | S | L | E | N | S | V | F |
| 83 | *Carnivora* | *Ailuropoda melanoleuca* | XP_002928483.1 | S | I | V | S | K | K | I | S | L | E | N | S | V | F |
| 84 | *Carnivora* | *Mustela erminea* | XP_032174508.1 | S | V | V | S | K | K | I | S | L | E | N | S | V | F |
| 85 | *Carnivora* | *Enhydra lutris kenyoni* | XP_022346413.1 | S | V | V | S | K | K | I | S | L | E | N | S | V | F |
| 86 | *Chiroptera* | *Rhinolophus sinicus* | XP_019573717.1 | S | I | I | S | S | K | L | S | L | E | Q | S | I | F |
| 87 | *Chiroptera* | *Artibeus jamaicensis* | XP_036990119.1 | R | I | I | S | K | K | I | S | L | E | N | S | V | F |
| 88 | *Chiroptera* | *Miniopterus natalensis* | XP_016071167.1 | S | I | I | S | K | K | I | S | H | E | N | S | V | F |
| 89 | *Chiroptera* | *Rhinolophus ferrumequinum* | XP_032949400.1 | S | I | I | S | N | K | L | S | L | E | Q | S | V | F |
| 90 | *Pholidota* | *Manis pentadactyla* | XP_036778635.1 | S | I | V | S | K | K | I | S | I | E | N | S | V | L |
| 91 | *Chiroptera* | *Myotis lucifugus* | XP_014318288.1 | S | I | M | S | S | K | I | S | L | E | T | S | V | F |
| 92 | *Chiroptera* | *Myotis myotis* | KAF6291666.1 | S | I | M | S | S | K | I | S | L | E | T | S | V | F |
| 93 | *Pholidota* | *Manis javanica* | KAI5940692.1 | S | I | V | S | K | K | I | S | I | E | N | S | V | L |
| 94 | *Chiroptera* | *Hipposideros armiger* | XP_019505159.1 | S | I | I | L | K | K | L | S | L | E | Q | S | V | F |
| 95 | *Chiroptera* | *Myotis brandtii* | XP_014402801.1 | S | I | M | S | S | K | I | S | L | E | T | S | V | F |
| 96 | *Carnivora* | *Suricata suricatta* | XP_029791667.1 | S | I | I | S | K | K | I | S | V | E | N | S | V | F |
| 97 | *Chiroptera* | *Pipistrellus kuhlii* | KAF6316575.1 | S | I | I | S | S | K | I | S | L | E | K | S | V | F |
| 98 | *Chiroptera* | *Eptesicus fuscus* | XP_027985151.1 | S | I | M | S | N | K | V | S | L | E | K | S | V | V |
| 99 | *Carnivora* | *Lontra canadensis* | ACM90097.1 | S | V | V | L | K | K | I | S | L | E | N | S | V | F |
| 100 | *Rodentia* | *Cavia porcellus* | XP_003466718.1 | R | L | T | - | P | S | V | V | V | N | R | S | Q | Q |
| 101 | *Carnivora* | *Ursus arctos* | XP_026343106.1 | R | L | T | - | P | T | I | L | T | N | R | A | Q | Q |
| 102 | *Artiodactyla* | *Bos indicus x Bos taurus* | XP_027384327.1 | T | W | - | T | I | I | W | T | - | T | S | S | S | T |
| 103 | *Primates* | *Sapajus apella* | XP_032116738.1 | T | L | T | - | P | T | I | V | T | N | R | S | Q | Q |
| 104 | *Chiroptera* | *Myotis davidii* | XP_006752907.1 | A | S | I | - | P | H | I | V | T | T | T | S | R | Y |
| 105 | *Carnivora* | *Meles meles* | XP_045839082.1 | S | V | V | S | K | K | I | S | L | E | N | S | V | F |
| 106 | *Carnivora* | *Mirounga leonina* | XP_034865837.1 | S | V | F | - | V | K | V | V | T | N | K | L | E | R |
| 107 | *Carnivora* | *Mirounga angustirostris* | XP_045732029.1 | R | L | T | - | P | T | V | V | T | N | R | S | Q | Q |
| 108 | *Carnivora* | *Ursus americanus* | XP_045640579.1 | R | L | T | - | P | T | I | L | T | N | R | A | Q | Q |
| 109 | *Carnivora* | *Neomonachus schauinslandi* | XP_021537593.1 | R | L | T | - | P | T | V | V | T | N | R | S | Q | Q |
| 110 | *Carnivora* | *Lynx rufus* | XP_046948572.1 | I | W | - | I | V | G | R | G | Q | N | R | I | S | Q |
| 111 | *Carnivora* | *Leopardus geoffroyi* | XP_045310949.1 | I | W | S | I | A | A | W | I | - | V | T | R | A | Q |
| 112 | *Carnivora* | *Prionailurus viverrinus* | XP_047696707.1 | S | W | I | S | I | I | W | S | D | S | T | R | E | W |
| 113 | *Carnivora* | *Puma concolor* | XP_025771056.1 | A | W | - | A | I | I | W | A | T | N | K | V | T | R |
| 114 | *Carnivora* | *Lutra lutra* | BAP05636.1 | R | L | T | - | P | T | V | V | T | N | R | S | Q | Q |
| 115 | *Carnivora* | *Panthera uncia* | XP_049490638.1 | V | W | - | V | I | I | W | V | R | Y | S | D | S | Q |
| 116 | *Primates* | *Pan troglodytes* | XP_024205121.1 | - | - | - | - | - | - | - | - | - | - | - | - | - | - |
| 117 | *Primates* | *Theropithecus gelada* | XP_025250591.1 | - | - | - | - | - | - | - | - | - | - | - | - | - | - |
